# Anæsthesia

**Published:** 1889-12

**Authors:** 


### Anæsthesia.

The subjects of selection and methods of use of anæsthetics, the danger of anæsthesia, and the treatment of the accidents which occur during it, have been so much discussed, indeed, so hackneyed, that we almost feel like apologizing for alluding to it. The matter is one, however, of the greatest importance, and the reporter of a case of death from ether, in the *New York Medical Journal* of May 18, held that the knowledge of physiological therapeutics has not yet been thoroughly diffused through the rank and file of the profession. In this case a middle-aged Frenchman who had been suffering from tubercular disease, with albuminous urine, and aortic and mitral systolic murmurs, and feeble heart-sounds, was given ether with the intention of the performance of Syme's amputation. A few moments after etherization was commenced the respiration faltered, and the patient became deeply cyanosed. This somewhat alarming condition quickly passed away, and five minutes later the assistant having the pulse under observation suddenly announced that it had ceased. "Immediately hypodermic injections of brandy, ether, and sulphate of atropine were given; amyl nitrite was applied to the nostrils, artificial respiration was practiced, and the head and shoulders were depressed by elevation of the foot of the table; but all in vain; the patient was dead."

According to the surgeon in charge, who reports the case, the death was the result of syncope, and largely the outcome of the diseased condition of the heart, which was found at the autopsy to have a completely adherent pericardium, atheroma of the aorta, and stenotic mitral valve. The kidneys were also in a condition of advanced contraction.

In the first place, it is essential that a case like this be not allowed to go upon record as one of death due to ether. We doubt very much the wisdom of attempting a severe surgical operation, like amputation of the foot, in a man who has disease of the kidneys, disease of the mitral and aortic valves, and the other numerous organic ills which affected this Frenchman. If an operation was justifiable at all, in such a case, both the surgeon and the patient should appreciate the excessive risks, and no therapeutist should charge the death against the anæsthetic. The peculiar greatness of the danger in the case becomes manifest when the selection of the anæsthetic is thought of. The condition of the kidneys strongly contra-indicated the use of ether; the clinical evidence which we now have being enough to demonstrate that chloroform is safer than ether in cases of renal disease, unless the heart-muscle has undergone secondary degeneration. This is probably because the amount of chloroform required to produce anæsthesia is so much smaller than that of ether that less strain is thrown upon the organ that eliminates it, hence the diseased kidneys are less irritated by chloroform than by ether. On the other hand, in the case under consideration, the condition of the heart evidently contra-indicated the use of chloroform; in fact, both ether and chloroform were contraindicated, and nothing but the greatest necessity could justify their use.

It is especially, however, to the treatment employed that we want to call attention, and we have no hesitation in affirming that if the patient had any chance of recovery at all, such chance was destroyed by the methods used for his relief. Suppose a doctor, having a case of opium-poisoning to deal with, were, for the purpose of relief, to administer morphine hypodermically, what would be the judgment of the profession as to the method? Evidently that it was a madness. But here we are told that hypodermic injections of ether and brandy were given. Probably almost every one in the profession would agree with us in believing that the use of hypodermic injections of ether was improper; possibly only a minority would agree to the statement that the administration of the brandy was almost, if not quite, as dangerous. Ether and alcohol, are, however, chemically, almost identical; physically, except in the matter of volatility, they are exceedingly alike, and in their relations to the human organism they seem to differ only in so far as one is more volatile, and therefore, more quick and more fugacious in its action than the other. Each primarily stimulates, and secondarily depresses the heart, and we believe that in accidents of etherization the administration of alcohol is equivalent to the administration of more of the poisonous agent. This conclusion rests also not simply upon *a priori* argument. As long ago as 1883, R. Dubois, in a series of experiments made upon the lower animals, found that the free administration of alcohol intensifies the influence of chloroform and lessens the minimum fatal dose. Certainly alcohol and ether are more nearly equivalent remedies, than are alcohol and chloroform.—*Therapeutic Gazette*, July, 1889.

